# Antibody‐Functionalized Copper Oxide Nanoparticles with Targeted Antibacterial Activity

**DOI:** 10.1002/open.202200241

**Published:** 2023-05-24

**Authors:** Jorge A. Ontiveros‐Robles, Francisca Villanueva‐Flores, Karla Juarez‐Moreno, Andrey Simakov, Rafael Vazquez‐Duhalt

**Affiliations:** ^1^ Department of Bionanotechnology Centro de Nanociencias y Nanotecnología Universidad Nacional Autónoma de México Km 107 carretera Tijuana-Ensenada Ensenada Baja California 22860 México; ^2^ Escuela Nacional de Medicina y Ciencias de la Salud Tecnológico de Monterrey Av. H. Colegio Militar 4700 Chihuahua Chihuahua 31300 México

**Keywords:** antibody bioconjugate, antibiotic, copper oxide nanoparticle, gram selectivity

## Abstract

Copper oxide nanoparticles (CuO‐NPs) were functionalized with specific antibodies to target their antibacterial activity against Gram‐positive or Gram‐negative bacteria. The CuO‐NPs were covalently functionalized to cover their surface with specific antibodies. The differently prepared CuO‐NPs were characterized by X‐ray diffraction, transmission electron microscopy and dynamic light scattering. The antibacterial activities of the unmodified CuO‐NPs and the antibody‐functionalized nanoparticles (CuO‐NP‐AbGram^−^ and CuO‐NP‐AbGram^+^) were determined for both Gram‐negative *Escherichia coli* and Gram‐positive *Bacillus subtilis* bacteria. The antibody‐functionalized NPs showed a differential increase of their antibacterial activity according to the specific antibody. The CuO‐NP‐AbGram^−^ in *E. coli* showed reduced half maximal inhibitory concentration (IC_50_) and minimum inhibitory concentration (MIC) values when compared with unfunctionalized CuO‐NPs. On the other hand, the CuO‐NP‐AbGram^+^ also showed reduced IC_50_ and MIC values in *B. subtilis*, when compared with non‐functionalized CuO‐NPs. Thus, the functionalized CuO nanoparticles with specific antibodies showed enhanced specificity of their antibacterial activity. The advantages of “smart” antibiotic nanoparticles are discussed.

## Graphical abstract

Copper oxide nanoparticles were covalently functionalized with specific Gram‐positive and Gram‐negative antibodies for targeted and differential antibacterial activity.

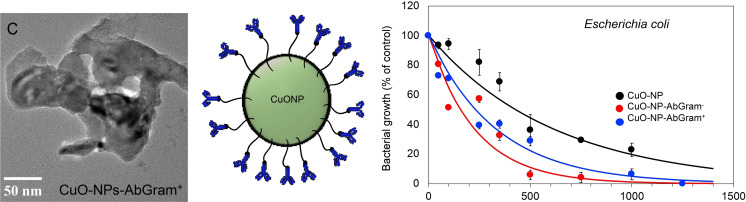

## Introduction

Bacterial resistance against antibiotics is one of the most critical global public health problems.^[1]^ The emergence of new multidrug‐resistant (MDR) bacteria compromises the effectiveness of antibiotics and leads to higher healthcare costs, more extended hospital stays, and increased morbidity and mortality.[^2^, ^3^] As a result of antibiotic‐resistant bacterial strains, 2.8 million people are infected, and more than 35,000 die each year in the USA.^[4]^ Although antibiotic resistance is a natural process that occurs from the interaction between microorganisms and their environment, it has increased exponentially, mainly due to the excessive and inappropriate use of antibiotics.^[5]^ In addition, research and development of new antibiotics have been held back due to the lack of incentives for the pharmaceutical industry, the high production costs, the long drug approval times, and the rapid appearance of MDR bacteria.[^6^, ^7^]

Due to the lack of new antibiotics and the presence of MDR, it is imperative to explore new alternatives. For centuries, metals such as silver (Ag), copper (Cu), and gold (Au) have been used for their antimicrobial properties.[^8^, ^9^, ^10^] These metals have been used for water disinfection and food preservation,^[11]^ to treat infections, and as orthopedic implants.^[12]^

In ancient times, copper was used against ailments and burns.^[13]^ The discovery of antibiotics has relegated the use of copper and other metals as antibiotics; however, with the increasing presence of MDR, the interest in the antibacterial properties of copper has increased.^[14]^ It is well known that the surface of copper‐based materials can kill Gram‐negative and Gram‐positive bacteria.^[13]^ The antibacterial activity of copper is mainly attributed to the release of ions (Cu^+^ and Cu^2+^) and the production of reactive oxygen species (ROS).^[15]^

Recently, with the rise of nanotechnology, nanomaterials based on metals have attracted attention as antibacterial agents. Specifically, copper oxide nanoparticles (CuO‐NP) have been highlighted as a bactericidal agent against diverse bacteria, such as methicillin‐resistant *Staphylococcus aureus* (MRSA), film‐forming bacteria, such as *Klebsiella pneumonia*, and *Enterococcus faecalis* bacteria associated with nosocomial diseases.[^16^, ^17^, ^18^] The antibacterial activity of CuO‐NPs can be attributed to the direct interaction between nanoparticles (NPs) and bacteria, followed by the release of Cu^2+^ ions caused by the dissolution of NPs and the induction of ROS. First, NP adsorption at the bacterial membrane reduces the transmembrane electrochemical potential. Second, sufficiently small nanoparticles and/or the released Cu^2+^ ions can permeate the cell membrane. Finally, NPs and Cu^2+^ ions induce ROS production, causing lethal changes in cells, DNA, and protein and lipid alterations.[^19^, ^20^]

Bioconjugates of gold NPs and antibodies have been used in an immunobiosensor.^[21]^ The biosensor based on gold nanoparticles (AuNPs) bioconjugated with anti‐*Escherichia coli* O157 : H7 antibodies was able to detect bacteria at a limit concentration of 100 cells/mL. In addition, the biosensor showed no significant changes (less than 3 %) when in the presence of *E. coli* DH5α, *E. coli* K12, and *Staphylococcus aureus*, confirming its selective specificity.^[21]^ On the other hand, Ivanova *et al*.^[22]^ developed bioconjugated zein nanocapsules with specific antibodies against *Staphylococcus aureu*s. The nanocapsules were loaded with a highly antibacterial oregano extract and their specific action and targeting were confirmed using a mixed culture of *S. aureus* and *P. aeruginosa*. The nanocapsules without antibodies showed a logarithmic reduction of up to 1.9 and 1.3 for *S. aureus* and *P. aeruginosa*, respectively. In contrast, the nanocapsules containing the specific antibodies induced a logarithmic reduction of up to 3 for *S. aureus*, while for *P. aeruginosa*, it was less than 0.5. Thus, targeted nanosystems have been shown to bind specifically to the target bacteria, thereby inducing a significantly enhanced bactericidal activity *in vitro*.^[22]^

Therefore, the aim of this work is to evaluate the bioconjugated CuO‐NP with Gram‐positive or Gram‐negative specific antibodies as specifically targeted antibacterial agents Our proposal combines an abundant material with antibacterial activity and the recognition capacity of antibodies to enhance both the antibiotic activity and selectivity.

## Results and Discussion

**Figure 1:**
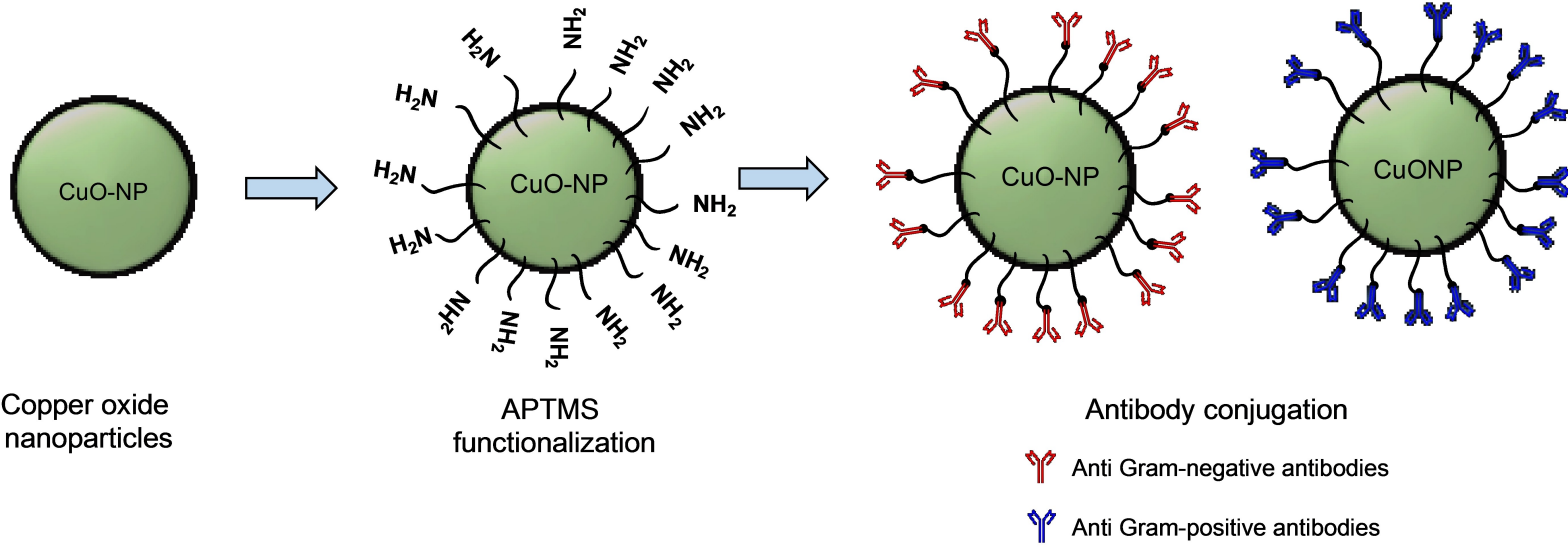
Schematic process for the production of antibody‐targeted CuO‐NPs.

**Figure 2:**
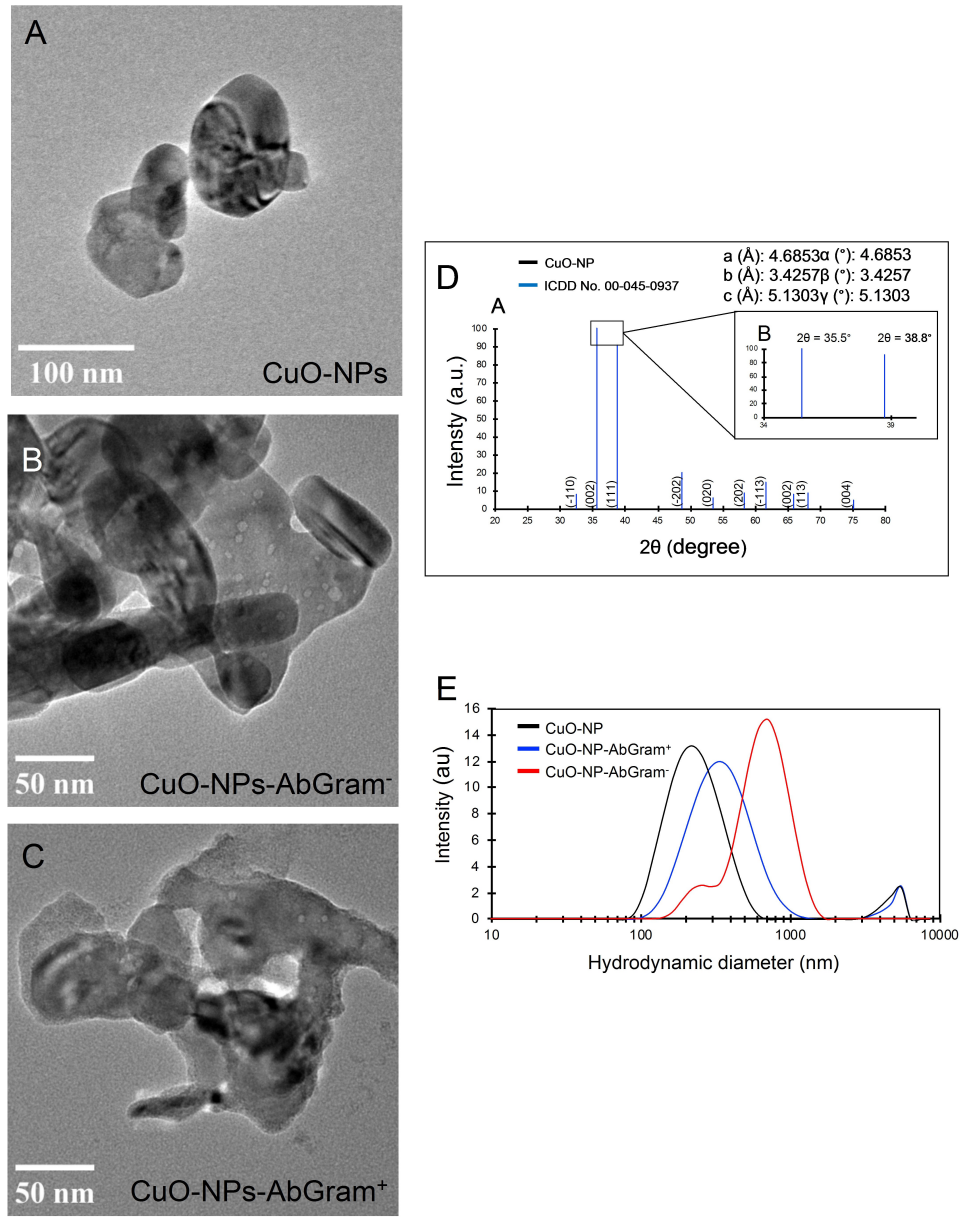
Images from transmission electronic microscopy (TEM) of the different preparations: A) Unfunctionalized CuO‐NPs, B) Gram‐negative antibody functionalized nanoparticles (CuO‐NP‐AbGram^−^), and C) Gram‐positive antibody functionalized nanoparticles (CuO‐NP‐AbGram^+^). X‐ray diffraction patterns (D) and hydrodynamic diameter measured by DLS.

Copper oxide nanoparticles were first functionalized with APTMS in order to have reactive NH_2_ groups on the nanoparticle surface. Then, two different antibody bioconjugates were obtained by covalently coupling monoclonal antibodies for Gram‐negative bacteria (CuO‐NP‐AbGram^−^) and Gram‐positive bacteria (CuO‐NP‐AbGram^+^) (Figure 1). The unmodified and modified nanoparticles were characterized by transmission electron microscopy (TEM), dynamic light scattering (DLS) and X‐ray diffraction (Figure 2).

The X‐ray diffraction patterns, analyzed by X'Pert Highscore, of the unmodified CuO‐NPs show a monoclinic crystalline phase (Figure 2c) according to the reference crystallographic card (ICDD: 00–045‐0937), where the most intense signals are located at 32.52°, 35.46°, 38.68°, 48.55°, 53.24°, 58.19°, 61.47°, 65.68°, 67.64° and 74.78°. These intensity peaks correspond to the crystalline planes (h k l): (−110), (002), (111), (−202), (020), (202), (−113), (022), (113) and (004).

The APTMS functionalization of CuO‐NPs and the antibody bioconjugation was successful as demonstrated by DLS (Table 1). The CuO‐NPs showed a negative zeta potential (−12.4 mV), while after amination (CuO‐NP‐NH_2_), the surface charge changed to a positive value (21.1 mV). After antibody bioconjugation, the nanoparticles showed a negative zeta potential (−17 and −18 mV) due to the protein covering.

**Table 1:** Zeta potential of the different CuO‐NP preparations.

Nanoparticle	Zeta potential [mV]
CuO	−12.4±4.0
CuO‐NP‐NH_2_	21.2±4.5
CuO‐NP‐AbGram^−^	−17.3±4.7
CuO‐NP‐AbGram^+^	−18.9±3.5

The unmodified CuO‐NPs showed a hydrodynamic diameter of 242±96 nm and a polydispersity index of 0.29 measured by DLS (Figure 2E), which is consistent with the size distribution determined by TEM (123±68 nm) (Figure 2A). As expected, the hydrodynamic diameter increased after the biocojugation with the antibodies to 240±42 nm for the CuO‐NP‐AbGram^+^ and 707±243 nm for CuO‐NP‐AbGram^−^. This increase could be partially due to aggregation produced after the unspecific chemical protein‐protein crosslinking, as supported by the TEM images (Figures 2B and 2 C). The antibody content in the functionalized NPs was estimated for CuO‐NP‐AbGram^+^ obtaining 1.43 μg of protein per mg of CuO nanoparticle.

**Figure 3:**
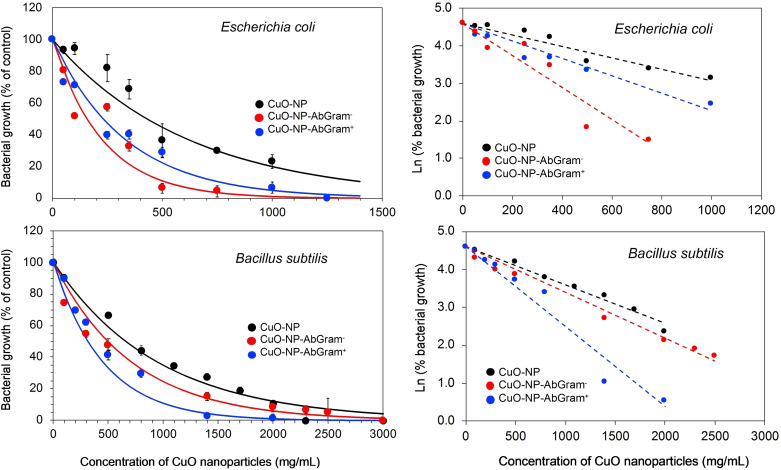
Bacterial growth at different nanoparticles concentration and with different copper oxide nanoparticles preparations. The growth inhibition was fitted a first order decay equation, linearized as logarithm of growth vs. CuO‐NP concentration.

The specificity of antibody‐conjugated nanoparticles was tested (Figure 3). *Escherichia coli* as Gram‐negative bacteria and *Bacillus subtilis* as Gram‐positive bacteria were assayed. In all the cases, the bacterial growth inhibition showed a first order decay behavior.

*E. coli* was more sensitive to the unmodified CuO‐NPs than *B. subtilis*. Functionalized nanoparticles with Gram‐negative antibodies (CuO‐NP‐AbGram^−^) reduced the IC_50_ for *E. coli* to 36 % of its value with unfunctionalized NPs, while the IC_50_ of *B. subtilis* showed a slight decrease to 69 % of the value with unfunctionalized nanoparticles (Table 2). On the other hand, functionalized nanoparticles with Gram‐positive antibodies reduced the IC_50_ of *B. subtilis* to 55 % of the value found with unfunctionalized CuO‐NPs, and the IC_50_ of *E. coli* is also reduced to 54 %, a similar value than the one obtained with CuO‐NP‐AbGram^−^. Optical microscopy images showed well‐dispersed bacterial cells in both preparations, and no hetero‐agglomeration of bacteria and nanoparticles was observed (see Supporting Information), assuring no underestimation of bacterial counts.

**Table 2:** Half maximal inhibitory concentration (IC_50_) and minimal inhibitory concentration (MIC) of different CuO‐NP preparations on Gram‐negative *E. coli* and Gram‐positive *B. subtilis*.

Nanoparticle	Bacteria	IC_50_ [μg/mL]	MIC [μg/mL]
CuO‐NP	*E. coli*	467±14	1250
	*B. subtilis*	681±11	2250
CuO‐NP‐AbGram^−^	*E. coli*	167±32	850
	*B. subtilis*	471±34	2900
CuO‐NP‐AbGram^+^	*E. coli*	254±14	1300
	*B. subtilis*	374±32	1600

The nanoparticles’ antibiotic specificity is also found in the minimal inhibitory concentration (MIC) measurements (Table 2). *E. coli* showed, again, to be more sensitive than *B. subtilis* when treated with unfunctionalized nanoparticles. The treatment with CuO‐NP‐AbGram^−^ fell to 68 % of the MIC for *E. coli* obtained with unmodified CuO‐NPs, while *B. subtilis* showed a higher MIC value. In contrast, the MIC value for *B. subtilis* was reduced to 71 % of the MIC value with non‐functionalized CuO‐NPs, while for *E. coli* no differences were found for the MIC values with unfunctionalized nanoparticles.

**Figure 4:**
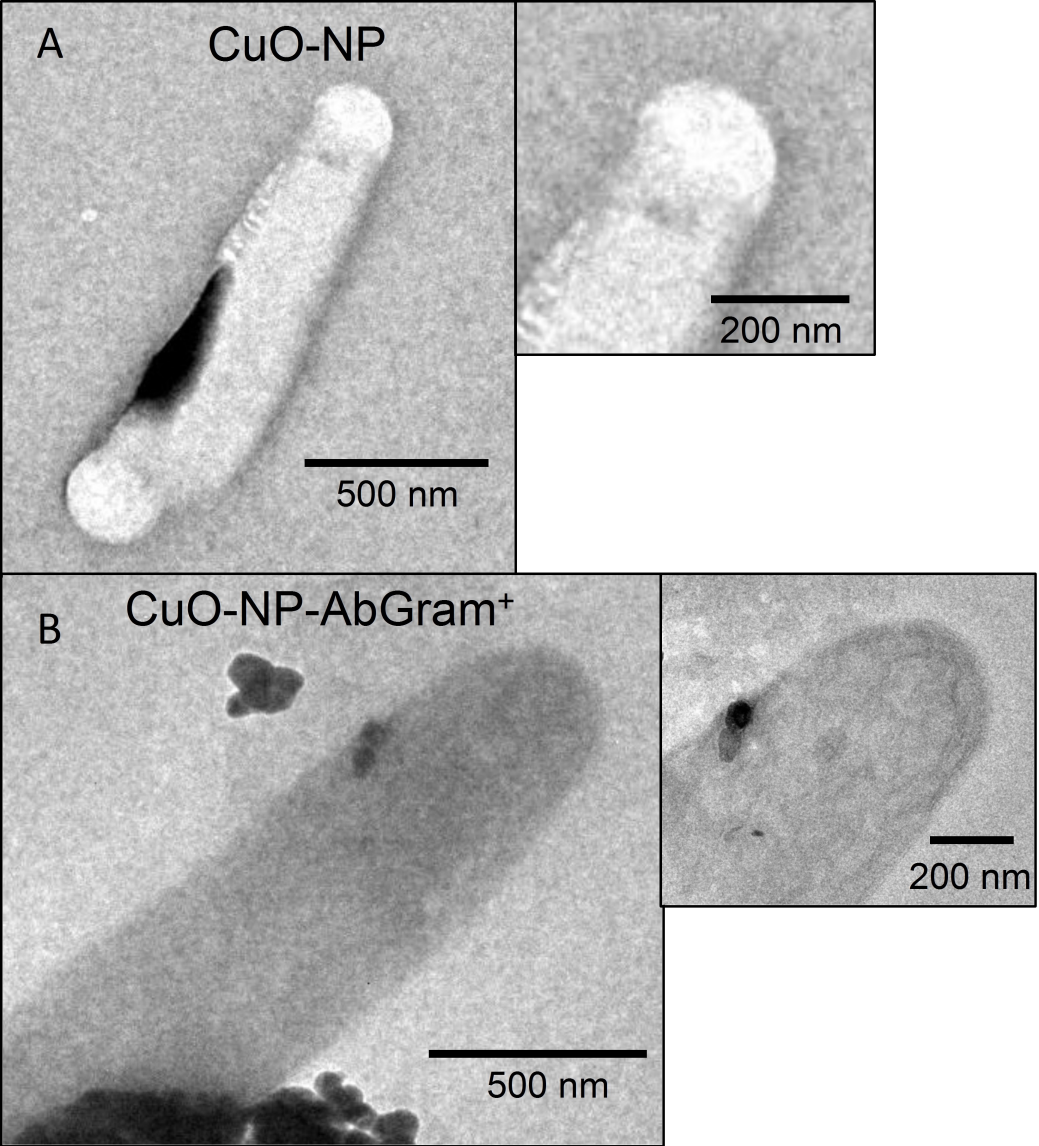
Transmission electron microscopy (TEM) images of (A) *B. subtilis* exposed to unfunctionalized CuO‐NPs, and (B) *B. subtilis* exposed to functionalized CuO‐NP‐AbGram^+^.

Transmission electron microscopy (TEM) showed several CuO nanoparticles tightly bound to the *B. subtilis* cell wall when bacteria were exposed to CuO‐NP‐AbGram^+^, and no copper nanoparticles adsorbed to the cell wall were detected when unfunctionalized CuO‐NP were used (Figure 4). It is important to point out that the unbound nanoparticles were eliminated by centrifugation at low rpm as mentioned in the Experimental Section.

The abundance of multidrug‐resistant bacterial infections and the decline in the discovery of new antibiotics is fueling the research on new innovative infection treatments. “Smart” drugs, including antibiotics, are needed. These “smart” antibiotics are assumed to only affect the pathogenic bacteria without disturbing the beneficial microbiome. Here, CuO‐NPs were targeted for specific antimicrobial activity by surface functionalization with Gram‐negative and Gram‐positive antibodies. TEM images clearly showed that nanoparticles functionalized with antibodies tightly bind to the bacteria cell wall (Figure 4), while unfunctionalized CuO nanoparticles do not. Antibodies are large glycoproteins able to recognize, with high specificity, foreign invading microorganisms by specifically binding to a pathogen's proteins or antigens, facilitating their neutralization and destruction. All antibodies share the same basic structure which consists of four polypeptide chains, two light chains (L chains) and two heavy chains (H chains), held together by disulfide bonds. These four polypeptide chains form a symmetrical molecule commonly depicted as having a “Y” shape and comprised of two identical halves, each bearing identical antigen binding sites. Antibodies bind reversibly to unique regions or epitopes within specific antigens through weak non‐covalent interactions which include hydrogen, ionic, hydrophobic, and van der Waals bonds. The strength or affinity of antibody binding is determined by the net force of weak interactions between a single antibody binding site and its epitope.

On the other hand, Gram staining differentiates bacteria by the chemical and physical properties of their cell walls. Gram‐positive cells have a thick layer of peptidoglycan in the cell wall that retains the primary stain, crystal violet. Gram‐negative cells have a thinner peptidoglycan layer that allows the crystal violet to be washed out on addition of ethanol.

Antibody targeting of nanoparticles for antibiotic application has been reported in the literature. Gold nanoparticles have been conjugated to antibodies specific to *Staphylococcus aureus* peptidoglycan.^[23]^ Similarly, silver nanoparticles have been conjugated with a specific antibody against *S. aureus* combined with laser‐induced bacterial damage.^[24]^ On the other hand, polyclonal‐antibody‐modified bismuth nanoparticles were tested to specifically target *Pseudomonas aeruginosa* to enhance the X‐ray irradiation‐based strategy that was used to kill bacteria.^[25]^ As far as we know, no published reports showing the specificity of antibody‐functionalized antibacterial CuO nanoparticles were yet available.

Copper oxide nanoparticles are gaining interest as antibacterial agents because of their easy and cheap production. CuO‐NPs can be produced by green processes and combined with diverse organic and biological components to improve their antibacterial activity; they release the copper ions faster when compared with the well‐studied silver nanoparticles.^[26]^ CuO‐NPs have been reported as toxic for a diversity of bacteria including beneficial, pathogen, multidrug‐resistant bacterial, and film‐forming bacteria.[^16^, ^17^, ^18^]

The antibacterial mechanism of CuO‐NPs is still not fully elucidated. However, it could be attributed to the copper ion release, induction of reactive oxygen species (ROS), and the direct interaction with the cell membrane.[^19^, ^20^, ^27^] The direct interaction of NPs with the cell membrane induces a reduction of the electrochemical potential. Then, the released Cu^2+^ ions, or very small CuO‐NPs, can cross the membrane, inducing ROS production and lethal cell damages on lipids, proteins and ADN.

CuO‐NPs size and morphology seems to be important for the bactericidal activity. For example, CuO‐NPs of 5, 45 and 900 nm reduced the cell viability to 97 %, 94 % and 36 % for *S. aureus*, and to 99.9 %, 98 % and 70 % for *E. coli*, respectively.^[19]^ The antibacterial differences were attributed to the interaction with the cell membrane, cell internalization, and the faster release of Cu^2+^ ions. On the other hand, the CuO‐NPs morphology also seems to play a role in the antibacterial activity.^[28]^

Different values of IC_50_ and MIC for CuO‐NPs are available in the literature. These values significantly vary according to the bacteria species, the determination procedure, nanoparticle size and oxidation state of copper. Values as low as 1 μg/mL for *Lactobacillus acidophilus* or 10 μg/mL for *Lactobacillus casei* have been reported.^[29]^ On the other hand, higher values of 1000 μg/mL were reported for *Pseudomonas corrugate*.^[30]^ Concerning the nanoparticle form, spherically shaped CuO‐NPs showed higher antibacterial property on Gram‐positive bacteria, whereas sheet shaped CuO‐NPs were more active on Gram‐negative bacteria.^[31]^ In addition, depending the CuO‐NP shape, the IC_50_ value against *Bacillus subtilis* could vary at about 140 μg/mL. The nanoparticle corona could also have effect on the antibacterial activity. Tryptophan‐coated CuO‐NPs showed a IC_50_ of 7.95 μg/mL, in which severe damage to the cell envelope was first detected, followed for a ROS production.^[32]^ Finally, copper oxidation state is also important for the antibacterial activity; ultrasmall Cu_2_O NPs showed IC_50_ values of 21.2 μg/L and 18.6 μg/mL for *Pseudomonas aeruginosa* and *Bacillus subtilis* strains, respectively.^[33]^

Independently of the variation of IC_50_ values in the literature, our results show a decrease of 45 % of IC_50_ with CuO‐NP‐AbGram^+^ in *B. subtilis*, and a decrease of 64.2 % in *E. coli* with CuO‐NP‐AbGram^−^ when compared with those obtained with non‐functionalized CuO‐NPs (Table 1).

## Conclusion

Our results clearly show a selective antibacterial activity (Figure 3) when the CuO‐NPs were functionalized with specific antibodies as supported by the IC_50_ and MIC values (Table 1). Because the Cu^2+^ ion release is one of the main mechanisms for cell toxicity, the Cu^2+^ concentration in the close surroundings of the bacterial cell could be higher when the antibody‐functionalized CuO‐NPs are recognized and attached to the bacterial cell than the suspended free CuO‐NPs (Figure 4). We can conclude so far that the antibiotic nanoparticles could be functionalized with specific antibodies or specific ligands to recognize specific bacteria. This is important in order to selectively kill the pathogenic bacteria without affecting the beneficial microsome.

## Experimental Section

### Materials

Gram‐positive monoclonal antibodies from Santa Cruz Biotechnology (BDI 380: sc‐57752) and Gram‐negative monoclonal antibodies from Novus Biologicals (NBP2‐53146) were obtained. *Bacillus subtilis* (ATCC 6051) as Gram‐positive bacteria and *Escherichia coli* (DH5a) as Gram‐negative bacteria were used. The compounds (3‐amino‐propyl)‐trimethoxysilane (APTMS), triethylamine (Et_3_N) and *N*‐hydroxy‐succinimide (NHS) were purchased from Sigma‐Aldrich (St. Louis MO). *N*‐(3‐dimethylaminopropyl)‐*N*‐ethyl carbodiimide hydrochloride (EDC) was obtained from Honeywell Fluka (Arizona, USA), while ethanol (99.9 %), toluene (99.9 %), monobasic potassium phosphate and dipotassium phosphate were provided by Fermont (Monterrey, Mexico). Phosphate saline buffer (PBS) was prepared using sodium chloride, potassium chloride, monobasic potassium phosphate, and dibasic sodium phosphate. Oxide copper nanoparticles (CuO‐NPs) were obtained from Sigma‐Aldrich (St. Louis, MO). Luria Bertani (LB) culture medium was purchased from Thermo Fisher Scientific (Göteborg, Sweden). All chemical reagents were used without additional purification.

### Chemical functionalization of CuO‐NP

Chemical functionalization of CuO‐NPs was performed by using APTMS.^[34]^ A total of 96 mg of CuO‐NPs were dispersed in 19.2 mL of toluene under ultrasonication (Ultrasonic bath Fisher Scientific model FS20). After 10 min, 240 μL de APTMS and 144 μL Et_3_N were added dropwise to the mixture under nitrogen atmosphere. The flask was sealed, and it was stirred for 6 h at room temperature. The suspension was centrifuged in a Thermo Scientific Heraeus Multifuge X1R centrifuge for 10 min at 4,500 rpm and the pellet was washed with ethanol three times. The solids obtained were dried at room temperature. The functionalized CuO‐NPs were named as CuO‐NP‐NH_2_.

### Bioconjugation of CuO‐NP‐NH_2_

The CuO‐NP‐NH_2_ surface containing free amino groups was further covalently conjugated with the monoclonal antibodies, anti‐Gram positive (Ab^+^) or anti‐Gram positive (Ab^−^). 24 mg of CuO‐NP‐NH_2_ were suspended in 1 mL of 50 mM phosphate buffer at pH 6.8. Then, monoclonal antibodies Ab^+^ (100 μg) or Ab^−^ (100 μg) were added dropwise. Subsequently, EDC and NHS as carboxyl activators were added at a final concentration of 10 mM and 20 mM, respectively, and the solution was kept under gentle agitation at room temperature for 2 h. Then, the solution was centrifuged at 6,000 rpm for 5 min and washed three times with phosphate buffer in order to remove unbound antibodies. Finally, the pellet was recovered, resuspended with 1 mL 50 mM phosphate buffer (pH 6.8) and stored at a temperature of 4 °C. Bioconjugated CuO‐NP‐NH_2_ with anti‐Gram positive were named CuO‐NP‐AbGram^+^ and with anti‐Gram negative were called CuO‐NP‐AbGram^−^. The amount of antibody covalently bounded to the CuO‐NP was estimated according to Oviedo *et al*.^[35]^.

### Nanoparticle characterization

The morphologies of CuO‐NP, CuO‐NP‐GramAb^+^ and CuO‐NP‐AbGram^−^ were analyzed by transmission electron microscope (TEM, JEOL JEM‐2010) operated at 200 kV, and the images were analyzed using the Digital Micrograph 3 (Gatan, Inc. Pleasanton, CA). The crystal phase was analyzed by X‐ray diffraction (XRD, X BRUKER D2 PHASER) using Cu Kα (λ=1.5418 Å) in the range of 10° to 80°. Absorption spectra of CuO‐NP were recorded by UV‐Vis spectrophotometry (Perkin Elmer Lambda 25 UV/Vis) in the wavelength range 200–700 nm. The hydrodynamic diameter size of CuO‐NP, CuO‐NP‐NH_2_, CuO‐NP‐AbGram^+^ and CuO‐NP‐Ab^−^ and surface charge were measured with dynamic light scattering (DLS) and Zeta potential was determined using a Zetasizer Nano ZS (Malvern Panalytical).

### Antibacterial Activity Tests

The antibacterial activity of CuO‐NP, CuO‐NP‐AbGram^+^, and CuO‐NP‐AbGram^−^ was evaluated in *E. coli* and *B. subtilis* bacteria cultures. The NPs preparations were ultrasonicated for 15 s at maximal power (Zgymzn YM‐1000, Shanghai, China) for suspension homogenization and avoiding nanoparticle aggregation. The minimum inhibitory concentration (MIC) and half‐maximal inhibitory concentration (IC_50_) were determined. The MIC is defined as the lowest antibiotic concentration of antibiotic that is capable of completely inhibiting visible bacterial growth under controlled *in vitro* conditions. On the other hand, IC_50_ is defined as the compound concentration required to inhibit the bacterial growth of 50 % of the population.

### MIC determination

The initial bacterial inoculum was made by taking 5 colonies of bacteria (*E. coli* or *B. subtilis*) from an agar plate and suspending them in 10 mL of LB broth and incubating for 24 h at 37 °C. Then, the inoculum was adjusted to a McFarland standard of 0.5, and serial dilutions were made until the bacterial density reached 1×10^4^ CFU/mL (working solution). The MIC determination was performed in a sterile flat‐bottomed 96‐well microtiter plate. A working volume of 200 μL per well was used for treatment; 100 μL of working solution and 100 μL associated to the treatments with different concentrations of NPs (CuO‐NP, CuO‐NP‐AbGram^+^ or CuO‐NP‐AbGram^−^). The NPs stock solution contained 24,000 μg/mL concentration in copper basis suspended in 500 μL of ultrapure water (type I) and was homogenized by ultrasonication. Blanks were performed similarly, substituting 100 μL of working solution with sterile LB medium. Finally, the plate was incubated for 24 h at 37 °C and under orbital shaking.

The bacterial growth was determined by measuring the absorbance at 600 nm. To avoid NPs interference in the absorbance measurements, 80 μL were taken from the surface for each well and transferred to a new plate on which the optical density (OD) measurement was performed.

### Transmission electronic microscopy (TEM)

One mL of bacterial cultures of *Bacillus subtilis* at stationary growth phase were exposed to MIC concentration of CuO‐NP or CuO‐NP‐AbGram^+^ nanoparticles for 1 h. The treated samples were centrifuged at 2,000 rpm for 2 min to remove the unabsorbed nanoparticles. The pellet was washed with distilled water and centrifuged again at 15,000 rpm for 25 min, and the pellet was resuspended in 0.5 mL of distilled water. Using a dropper, 1 drop of cell suspension was deposited on carbon‐coated grids (80 mesh square grid, EMS, TED PELLA, Inc., Redding, CA, USA) and incubated for 2 min. The excess sample was removed from the grid using blotting paper. To stain the samples, 5 μL of 1 % uranyl acetate was added onto the grid and incubated for about 1 min. Finally, the grids were analyzed using transmission emission microscopy (TEM, JEOL‐2010, JEOL) operated at 200 kV.

### Statistical analyses

Each treatment was assayed at least in triplicate. Means, standard deviations and IC_50_ were calculated using the Microsoft Excel 2016 program. A two‐way analysis of variance (ANOVA) was used to compare values from different treatments. When significant differences among treatments were found (p≤0.01), Tukey's multiple comparison analysis was performed using the GraphPad Prism 8 program.

## Conflict of interest

The authors declare no conflict of interest.

1

## Supporting information

As a service to our authors and readers, this journal provides supporting information supplied by the authors. Such materials are peer reviewed and may be re‐organized for online delivery, but are not copy‐edited or typeset. Technical support issues arising from supporting information (other than missing files) should be addressed to the authors.

Supporting Information

## Data Availability

The data that support the findings of this study are available from the corresponding author upon reasonable request.
