# Supplementary material for: Antibody‐Functionalized Copper Oxide Nanoparticles with Targeted Antibacterial Activity
Source: ChemistryOpen. 2023 May 24;12(5):e202200241. doi: 10.1002/open.202200241 (PMC10209517; doi:10.1002/open.202200241)
Supplement: Supplementary file 1 — Supporting Information [file OPEN-12-e202200241-s001.pdf]

ChemistryOpen

Supporting Information

Antibody-Functionalized Copper Oxide Nanoparticles with Targeted Antibacterial Activity

Jorge A. Ontiveros-Robles, Francisca Villanueva-Flores, Karla Juarez-Moreno, Andrey Simakov, and Rafael Vazquez-Duhalt*

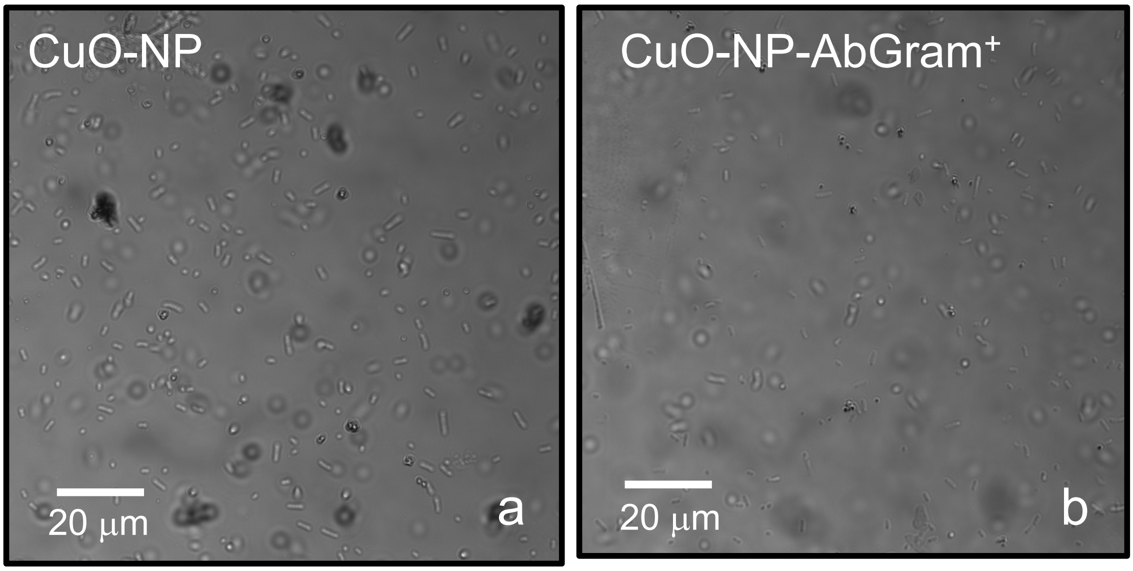

Figure SI. Images from optical microscopy of (a) *Bacillus subtilis* in the presence of unfunctionalized CuO nanoparticles and (b) *Bacillus subtilis* in the presence of CuO nanoparticles functionalized with antibodies anti-Gram positive. The bacterial cells are well dispersed in both preparations, and no hetero-agglomeration of bacteria and nanoparticles was detected. Few nanoparticle clusters are observed but without associated bacteria cells. Preparations were analyzed in a Nikon Ti microscope with a Spinning Disk de Yokogawa module and TIRF.
